# Accuracy of Preoperative 3D vs 2D Digital Templating for Cementless Total Hip Arthroplasty Using a Direct Anterior Approach

**DOI:** 10.1016/j.artd.2023.101260

**Published:** 2023-11-04

**Authors:** Thomas Aubert, Giacomo Galanzino, Philippe Gerard, Vincent Le Strat, Guillaume Rigoulot, Luc Lhotellier

**Affiliations:** Orthopedic Department, Diaconesses Croix Saint Simon Hospital, Paris, France

**Keywords:** Arthroplasty, Hip, Planning, Anterior approach, Cementless prosthesis

## Abstract

**Background:**

An important aspect of preoperative planning for total hip arthroplasty is templating. Although two-dimensional (2D) templating remains the gold standard, computerized tomography (CT)-based three-dimensional (3D) templating is a novel preoperative planning technique. This study aims to compare the accuracy of a 2D and 3D plan using an anterior approach for the placement of the same uncemented prosthesis.

**Methods:**

Two consecutive cohorts of 100 patients each were retrospectively analyzed. We analyzed the accuracy of the size of the implant (stem, cup, head), the length of head, and offset. As a secondary criterion, we analyzed the rates of stems with more than 3° of varus, fracture, and/or subsidence at 3 months postoperatively.

**Results:**

Within the exact size, the accuracy of the stem and cup size with the 2D plan was 69% and 56%, respectively. With the 3D plan accuracy being 88% (*P* = .0046) and 96% (*P* < .0001), respectively. Regarding size and length of the implant head, accuracy was 86% and 82% with the 2D plan and 100% (*P* < .0001) and 94% (*P* = .016), respectively, with the 3D plan. The offset of the implants increased beyond 3 mm in 23% of patients in the 2D group and in 5% of patients in the 3D group (*P* = .0003). The rate of varus stems was 10% in the 2D group and 2% in the 3D group (*P* = .03). Two fractures and one case of subsidence occurred in the 2D group. None were identified in the 3D cohort.

**Conclusions:**

A CT-based 3D plan is more accurate for implant size selection, allows better prosthetic offset, and reduces the rate of varus stems.

## Introduction

Total hip arthroplasty (THA) is a very successful surgery [1]. Innovation has led to better customization of implant positioning to restore the biomechanics of the hip [2,3] and ensure the success of long-term fixation [4]. Preoperative planning allows the optimal implant size, position, and possible difficulties [5] to be predicted. It reduces the risk of complications such as impingement, fracture, leg length difference, and dislocation, which can be responsible for patient dissatisfaction [6,7].

Templating is another important stage of preoperative planning for THA. Radiographs and computerized tomography (CT) scans help to anticipate the position and size of implants with precision [8]. Leg length and offset are recognized as important factors to improve the satisfaction of patients and avoid the risk of instability and trochanteric pain [9,10].

Traditional radiography has been substituted with digital radiography, allowing for greater accuracy [11]. Although two-dimensional (2D) templating remains the gold standard, three-dimensional (3D) templating based on CT scans is a new approach which is likely to improve accuracy in the preoperative templating of implants [[12], [13], [14], [15], [16]].

The placement of cementless implants depends on the bone density and can be very difficult to evaluate with the use of an anteroposterior radiograph. Cementless implants need underreaming and a perfect press-fit technique to avoid malposition, especially varus. Moreover, the risk of fracture or subsidence is directly related to the size of the stem [7].

The aim of this study is to compare the accuracy of a 2D digital plan with that of a 3D CT constructed plan in selecting the size of implants and the prosthetic offset of the same uncemented prosthesis using an anterior approach. As a secondary criterion, we analyzed the rates of varus stems, fracture, and/or subsidence at 3 months postoperatively.

## Material and methods

### Study design and participants

#### Cohort

Two consecutive cohorts of 100 patients each were retrospectively analyzed. The patients underwent total hip replacement in the same hospital between November 1, 2021, and December 1, 2022. There was no significant difference between the 2 groups in terms of age, sex, body mass index, or implant size (Table 1).

**Table 1:** Baseline characteristics of patients.

Baseline characteristics	Population 3D plan	Population 2D plan	*P* value
	n = 100	N = 100	
Age (years), mean (range)	63 (45-85)	66 (46-85)	.25
Male sex, No. (%)	39 (39%)	50 (50%)	.15
Side right, No (%)	51 (51%)	50 (50%)	.15
Height (cm), mean (range)	168 (151-186)	168 (150-180)	.595
Weight (kg), mean (range)	74.6 (43-107)	74.0 (46-120)	.72
BMI (kg/m^2^), mean (range)	22.1 (14.2-32.3)	21.8 (17.4-37.4)	.56

BMI, body mass index.

THA was performed in all patients using a cementless straight quadrangular stem and ceramic-on-ceramic head and liners (Meije Dynacup; Corin, Cirencester, UK). X-ray and CT scans were used to preoperatively plan the sizes of the implant components (ie, cup, stem, and head) using MediCAD 2D (Hectec GmbH, Altdorf, Germany) and 3D OPSInsight software (Corin, Cirencester, UK). All operations were performed by 3 surgeons using a direct anterior approach (DAA) to the hip with the patient in a supine position on a traction table without fluoroscopy.

The objective was to restore the femoral offset and the height of the femoral head center from the top of the greater trochanter. For the femoral head, 5 lengths could be used: −4 mm, −3.5 mm, 0, +3.5 mm and +4 mm. The cup was placed at the inferior teardrop and less than 2 mm lateral from Kohler's line, with an inclination between 35° and 50°. The suited position of the stem was with less than 3° of valgus or varus and with sufficient cortical contact or canal fill in the metaphyseal-diaphyseal junction [11].

The radiological analyses were assessed twice by 2 different examiners.

This study was approved by the local ethics committee, and the patients provided informed consent.

### Templates

The MediCAD software system (mediCAD; Hectec GmbH, Altdorf, Germany) was used for digital 2D preoperative planning. Templates with the same magnification coefficient (1.15) were used for the 2D preoperative planning, and the planned components were drawn on the films.

**Supplemental Figure 1:**
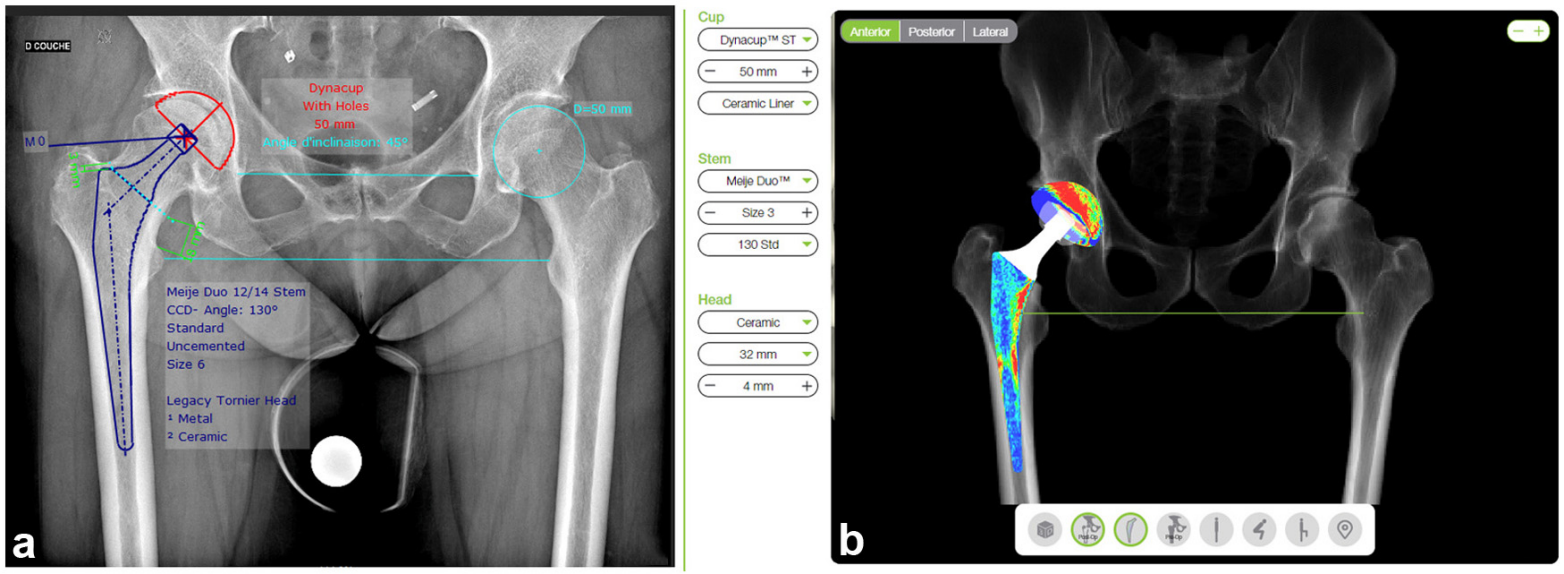
Preoperative 2D and 3D templating. (a) Digital 2D preoperative planning. (b) Three-dimensional preoperative planning.

Optimized Positioning System (OPSInsight; Corin, Cirencester, UK) was used for the preoperative 3D plan. The cup was placed relating to the posterior and anterior acetabular walls in addition to the supero-lateral acetabular margin. The stem size was selected to maximize both fill and fit in the metaphysis. The final cranial-caudal position of the stem can be reached when this implant is blocked. The femur was planned in an anatomic position, respecting native anteversion (Supplemental Fig. 1).

### Outcome

Accuracy was calculated postoperatively by comparing the number of implants that were the same size as the template size, within 1 (±1) or 2 (±2) size differences.

We compared the size of the head between 28 mm and 36 mm, which was used to match the size of the cup (28 mm for a cup under 44 mm, 32 mm for a cup between 46 and 50 mm, and 36 mm for a cup over 52 mm).

The head length was divided into −4 mm, −3.5 mm 0, +3.5 mm and +4 mm and was planned to reproduce the anatomical offset.

The angulation of the neck was divided into standard, 130°, and lateralized, 127°. The choice of angulation was made to reproduce the anatomical offset as well.

The offset of the neck is the same for stem sizes 1 to 3 (28.7 mm for the standard stem and 32.7 mm for the lateralized) but changes for sizes 4 to 6 (30.6 mm for the standard stem and 34.8 mm for the lateralized) and for sizes 7 to 9 (32.6 mm for the standard stem and 36.9 mm for the lateralized).

The global implant offset was analyzed using the offset of the neck with respect to the size of the stem, angulation of the neck, and head length. The change in offset was defined as a change of more than 3 mm between the planned offset and the actual offset of the implant.

Varus malalignment was defined as a deviation of more than 3° between the femoral and stem axes [17].

Subsidence was defined as a vertical migration >3 mm between the tip of the greater trochanter and shoulder of the prosthesis on the 3-month postoperative radiographs [18].

### Data analyses

Continuous variables are described by using means and ranges. We compared means and proportions between these groups by using Student’s *t* tests, analyses of variances (Mann–Whitney tests), or Fisher’s exact tests (when appropriate).

The group size was determined to be 99 to allow a 5% difference in the rate of implant size or varus stems according to the type of plan, with 85% power and 5% alpha risk.

All analyses were performed using R (version 4.0.0, R Foundation for Statistical Computing, Vienna, Austria; https://www.R-project.org/). Two-tailed *P* values <.05 were considered significant.

## Results

### Planned implantation vs actual implantation with the 2 methods

There was a statistically significant difference in the planned and actual implant cup size between the 2D (*P* < .0028) and 3D groups (*P* = .00705) as well as in the planned and actual stem size between the 2D (*P* = .0046) and 3D groups (*P* = .0172) (Table 2). The differences in the head length and size were statistically significant in the 2D group (*P* < .0001 and *P* = .0001) but not in the 3D group (*P* = .1 and *P* = 1) (Table 2).

**Table 2:** Planned implantation vs actual implantation with the 2 methods.

Implant parameters	Planned	Implanted	*P* value
	n = 100	n = 100	
Stem size			
2D plan, size (%)	1 (1), 2 (3), 3 (23), 4 (23), 5 (18), 6 (17), 7 (9), 8 (4), 9 (2)	1 (1), 2 (8), 3 (25), 4 (16), 5 (22), 6 (11), 7 (12), 8 (5)	.0046
3D plan, size (%)	1 (3), 2 (11), 3 (18), 4 (31), 5 (14), 6 (15), 7 (6), 8 (2)	1 (3), 2 (6), 3 (23), 4 (27), 5 (18), 6 (14), 7 (7), 8 (2)	.0172
Cup size (mm)			
2D plan, size (%)	44 (5), 46 (10), 48 (19), 50 (17), 52 (24), 54 (17), 56 (5), 58 (2), 60 (1)	44 (3), 46 (15), 48 (20), 50 (19), 52 (25), 54 (15), 56 (2), 58 (1)	.0028
3D plan, size (%)	44 (1), 46 (11), 48 (14), 50 (26), 52 (27), 54 (10), 56 (8), 58 (2), 60 (1)	44 (1), 46 (12), 48 (15), 50 (24), 52 (29), 54 (9), 56 (7), 58 (3)	.0075
Head length (mm)			
2D plan, length (%)	−4 (2), −3,5 (1), 0 (87), +3,5 (0), +4 (10)	−4 (9), −3,5 (1), 0 (76), +3,5 (0), +4 (14)	.358
3D plan, length (%)	−4 (16), −3,5 (1), 0 (62), +3,5 (0), +4 (21)	−4 (19), −3,5 (1), 0 (59), +3,5 (0), +4 (21)	.103
Head size (mm)			
2D plan, size (%)	28 (5), 32 (46), 36 (49)	28 (3), 32 (54), 32 (43)	.109
3D plan, size (%)	28 (1), 32 (51), 36 (48)	28 (1), 32 (51), 36 (48)	1

### Accuracy of implant size prediction based on 2D and 3D plans

**Figure 1:**
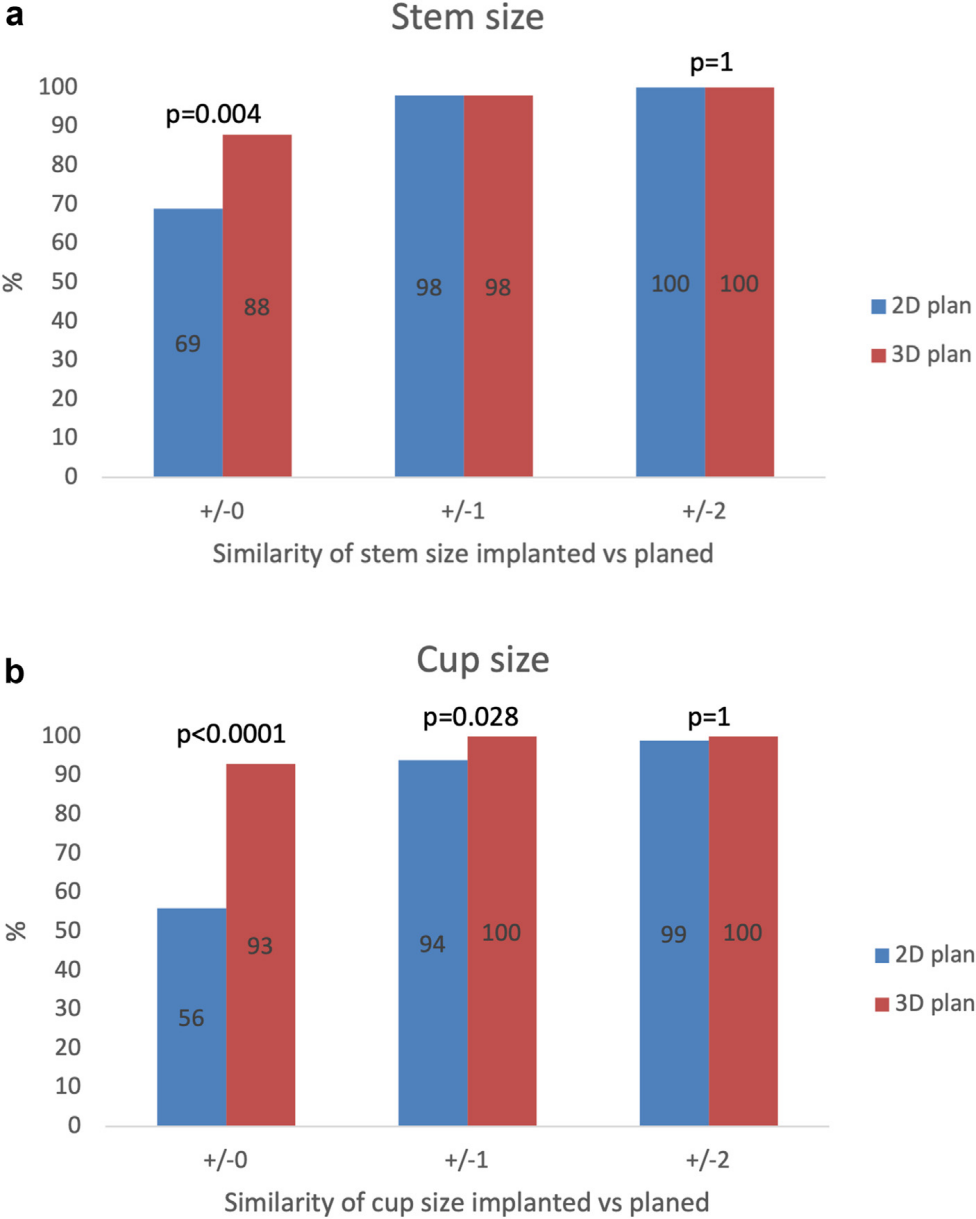
(a) Two-dimensional plan (2D) vs three-dimensional plan predicted cup size difference from final size implanted. (b) Two-dimensional plan vs 3D plan predicted stem size difference from final size implanted.

Within the exact implant size, the accuracy of the stem and cup size with the 2D plan was 69% and 56% while that with the 3D plan was 88% (*P* = .0017) and 96% (*P* < .0001), respectively (Table 3). Within one size, the accuracy of the stem and cup size with the 2D plan was 98% and 94% while that with the 3D plan was 98% (*P* = 1) and 100% (*P* = .028), respectively, (Fig. 1).

**Table 3:** Accuracy of implant size prediction based on 2D and 3D plans.

Implant parameters	2D plan	3D plan	*P* value
	n = 100	n = 100	
Stem size			
±0	69%	88%	**.0046**
±1	98%	98%	1
±2	100%	100%	1
Cup size			
±0	56%	93%	**<.0001**
±1	94%	100%	**.028**
±2	99%	100%	1
Head			
Exact length	82%	94%	**.016**
Exact size	86%	100%	**<.0001**
Stem			
Exact neck angulation	96%	100%	.12
Exact neck offset	89%	97%	**.0048**
Global implant’s offset			
Offset change	28%	8%	**.0003**

Bold values denote statistical significance at the *P* < .05 level.

**Figure 2:**
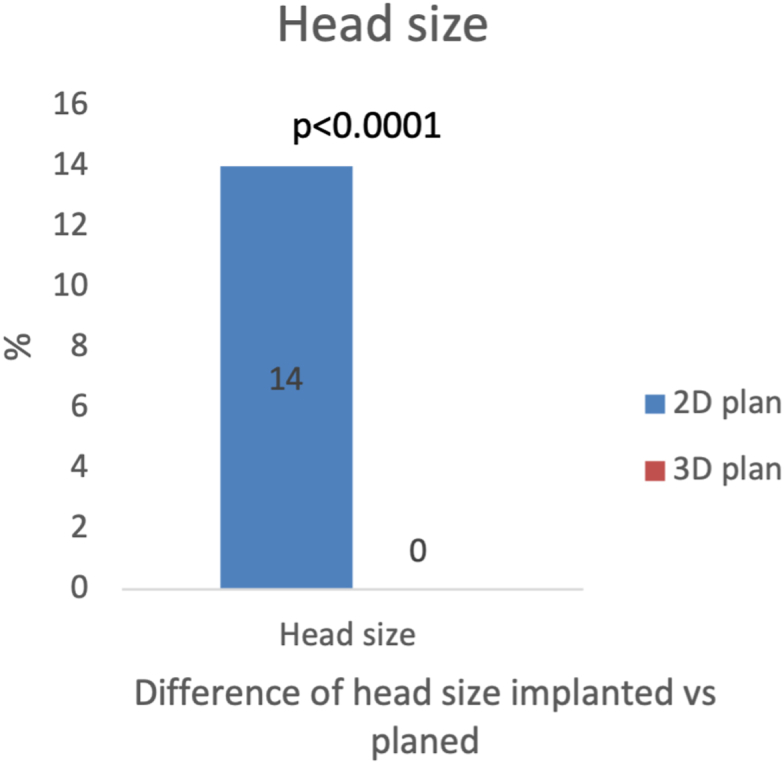
Two-dimensional plan vs 3D plan predicted head size difference from the final size implanted.

Regarding the head, the exact length and size of the implants implanted were 82% and 86% with the 2D plan and 94% (*P* = .016) and 100% (*P* < .0001) with the 3D plan, respectively, (Fig. 2).

Concerning the angulation of the neck for implantation, the actual angle vs the planned angle (130° vs 123°) was not significantly different between the 2 groups, 96% vs 100% (*P* = .12), but the offset was the same in 89% of the 2D group compared to 97% in the 3D group (0.0048).

**Figure 3:**
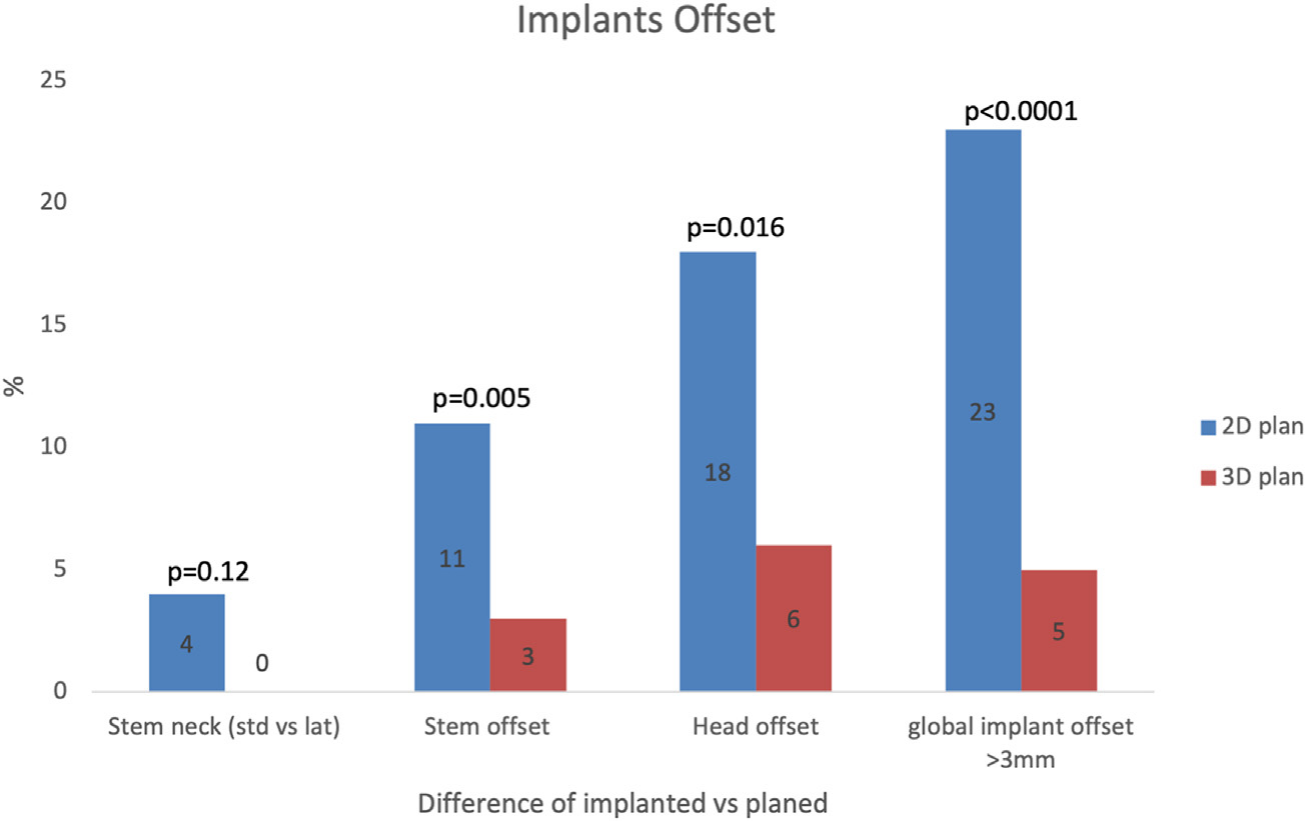
Two-dimensional plan vs 3D plan predicted implant offset difference from final offset executed.

Finally, the global offset of the implants changed by more than 3 mm in 23% of the patients in the 2D group and in 5% of the patients in the 3D group (*P* = .0003) (Fig. 3).

### Varus, fracture, and subsidence at 3 months postoperatively

The rate of varus stems was 10% in the 2D group and 2% in the 3D group (*P* = .03).

Two intraoperative fractures and 1 case of subsidence occurred at 3 months in the 2D group, while no fractures or subsidence identified in the 3D group.

## Discussion

Preoperative templating is a very important stage in the successful implantation of a prosthesis [5].

In a recent study, the CT-based template accurately predicted the position in 38% of cases involving the acetabulum and in 35% of cases involving the femur [19], and when cases within 1 size of the actual implant were included, the accuracy improved to 80% for the acetabulum and 85% for the femur using a 3D template. In our study, we showed that the accuracy of the stem and cup size was 88% and 94% with the 3D plan, which was significantly higher than that of the 2D plan (69% and 56%, respectively). Our result seems better within the exact size using a DAA and concordant within one size to the other studies [20,21]. In one study, the researchers aimed to compare 2D and 3D plans using an anterior approach, but with only one surgeon, and with different stems in the 2 comparative groups of 30 patients, with a prediction rate for the stem and the cup sizes, respectively, of 100% and 96% in the 3D group [16]. Our study compared 2 comparative groups that received the same cup and stem to analyze only the accuracy of the plan.

**Figure 4:**
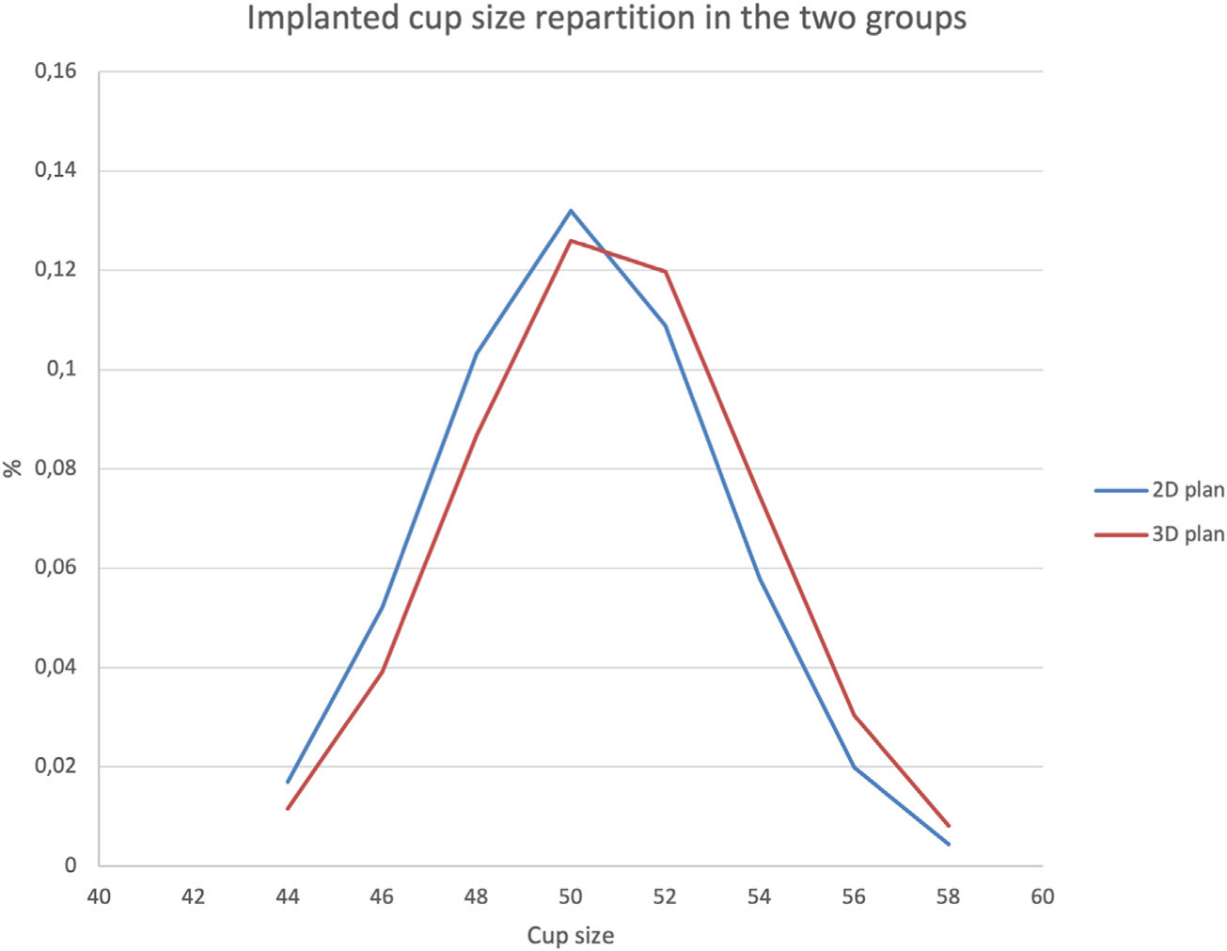
Gaussian curves of the cup sizes implanted in the 2 cohorts.

The head size was shown to be more accurate using the 3D plan (100% vs 86%, *P* < .0001), which is important for reducing the risk of instability or impingement caused by using a smaller size [22,23]. The majority of cups that were implanted were between 50 and 52 mm (Fig. 4), and the size of the head between those 2 sizes of cups was 32 vs 36 mm. As the accuracy of the exact size of the implanted cup under the 3D plan was higher, the accuracy of the exact size of the head implanted was higher.

**Figure 5:**
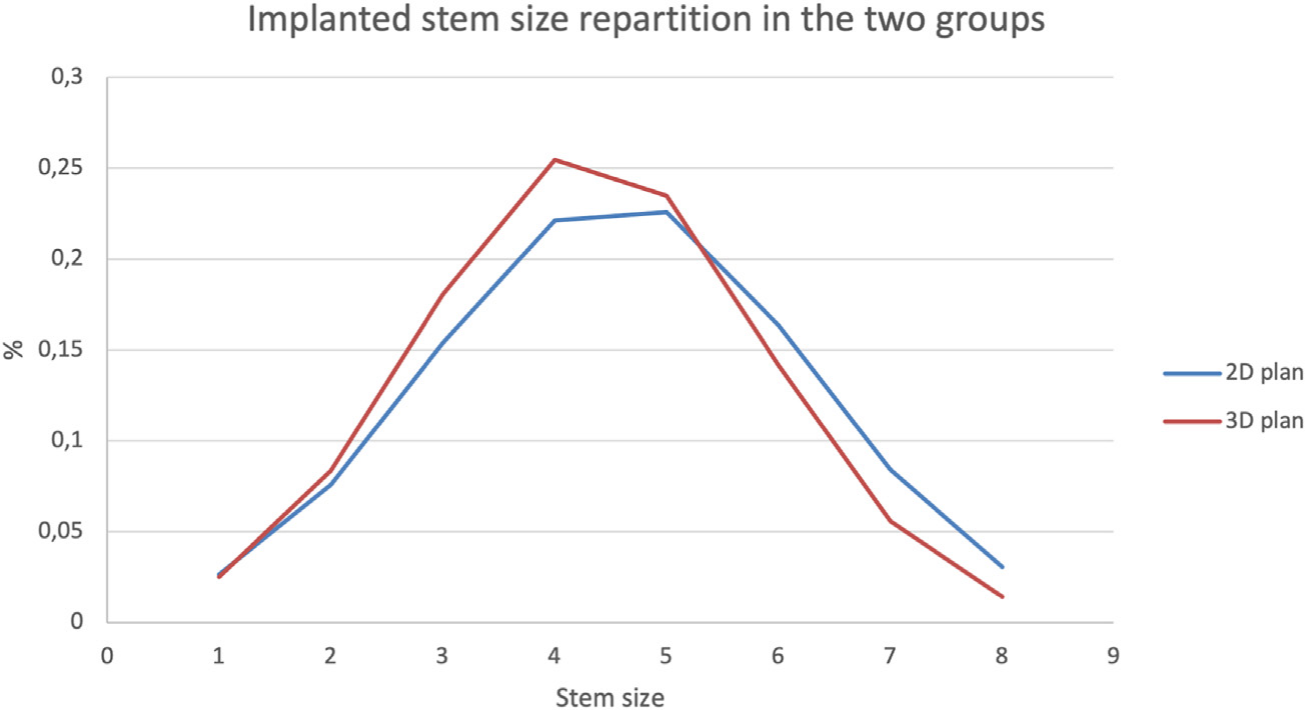
Gaussian curves of the stem sizes implanted in the 2 cohorts.

The prosthetic offset changes with respect to the neck of the stem (standard vs lateralized), the head length, and the size of the stem. In our study, the global offset changed by more than 3 mm in only 5% of patients in the 3D group vs 23% of patients in the 2D group. The selected plan can lead to significant changes in the offset and poorer outcomes (instability or trochanteric pain) [9,10]. This variability of the offset is mainly due to 2 different parameters. First, size 3 and 4 stems are most commonly implanted in both groups and the neck offset change between these 2 sizes (1.9 mm of difference) (Fig. 5). It could be interesting to alter the offset of those implants to maintain consistency among implant sizes. Second, the length of the head was accurately selected for 94% of the patients in the 3D group and for 82% of the patients in the 2D group; both of these rates were higher than the 64% reported in a previous study [20]. Changing the head length could restore the global offset in cases in which stems of different sizes are implanted. It has been shown to be safe in revision and reoperation and should be performed to optimize hip stability and the center of rotation [24]. However, changing the length of the head was an effective means for adapting to a different size stem and restoring the native offset in only 16.7% of cases. These findings might support the idea that surgeons mostly changed the lengths of the head to achieve a desired leg length rather than a desired offset. The system using the 3D plan provides a femoral cutting block that has been shown as having a precision of 2 mm in the cut of the neck [25]. This should allow greater confidence when positioning the stem during surgery, reduce the need to compromise, and therefore lead to a lower rate of change in the head length.

It is evident that the imprecision in surgery can cause additional offset variability. We only analyzed the change in offset for the implants but not their position, due to the difference in accuracy between X-ray and CT scans. The mediolateral position of the cup is one of the most important factors changing the offset, as the mean displacement of the hip center of rotation was 5 mm medially in a recent study [26]. However, the use of fluoroscopy can help to avoid malpositioning [27].

Furthermore, even if a varus stem does not affect the long-term functional outcome in patients undergoing cementless THA [28,29], the position of the stem in the frontal plane can change the offset by 1.5 mm for every degree of varus [30]. When taking the DAA, the risk of varus stem alignment was 59% when a straight tapered stem was used [31], with 9% of those stems having more than 3° of varus. In our study, the risk of the stem having more than 3° of varus was higher in the 2D group (10%) than that in the 3D group (2%) and could ultimately lead to an increase in offset. Regarding the 2D plan, this result can be explained by the use of a smaller prosthesis than originally templated to reduce the risk of periprosthetic fracture or leg lengthening associated with oversized prostheses [7], but undersized stems increase the risk of varus stems. The use of the 3D plan helps the surgeon to plan the right size and avoid an increased risk of revision and varus due to the use of an undersized stem [32], which was less important than expected, indicating the interest in the 3D plan to avoid varus stems even if using a DAA. We acknowledge that varus angulation is not always a technical error and that having a proximal varus anatomy could be a predisposing factor for such an occurrence [33] and was not analyzed in our study. However, a CT scan can evaluate a potential varus alignment preoperatively to allow proper adaptation of the stem size.

Due to the small size of our cohort, no fractures were found in the 3D group, whereas 2 fractures were found on the day of surgery in the 2D group. Because the 3D plan was more accurate for selecting the right stem size, the risk of fracture associated with the use of an oversized stem was avoided.

The limitations of this study are worth mentioning. All the operations were performed by 3 different surgeons; however, several studies have shown that 3D computer-based preoperative planning is more advantageous than traditional template planning independent of surgeon experience [34], leading to biases in estimating the reliability of 2D and 3D templating.

All of the stems were tapered straight with a quadrangular cross-section and fully coated, so it would be interesting to compare the accuracy of the plan using different geometries, textures, and stem coatings.

Moreover, the use of intraoperative planning with a predetermined implant size may introduce a bias in the final size implanted; however, the planning was available with both methods.

Furthermore, 3D templating was significantly more accurate for implant size selection and for achieving offset and avoiding varus in our study, but it is still difficult to determine if it has any clinical benefit. Compared to 2D templates, 3D templates are less accessible, more time-consuming to create, more irradiating even with the use of a low-dose CT scan [25], and more expensive; thus, studies analyzing the cost/benefit ratio are needed [7].

## Conclusions

A CT-based 3D plan is more accurate for implant size selection, which allows for better prosthetic offset and more accurate head size selection in addition to reducing the incidence of varus stems.

## Conflicts of interest

The authors received no financial or material support for the research, authorship, and/or publication of this article. T.A. and G.R. are consultants for Corin.

For full disclosure statements refer to https://doi.org/10.1016/j.artd.2023.101260.

## Credit authorship statement

G.G. contributed to formal analysis and validation. T.A. contributed to conceptualization, data curation, formal analysis, investigation, methodology, supervision, validation, writing – original draft, and writing – review & editing. G.R., V.L.S., and L.L. carried out validation. P.G. contributed to investigation and validation.

## Appendix A Supplementary data

Conflict of Interest Statement for Galanzino

Conflict of Interest Statement for Lhotellier

Conflict of Interest Statement for Gerard

Conflict of Interest Statement for Aubert

Conflict of Interest Statement for Le Strat

Conflict of Interest Statement for Rigoulot
